# Successful treatment for severe pancreatitis with colonic perforation using video-assisted retroperitoneal debridement: A case report

**DOI:** 10.1016/j.ijscr.2018.09.046

**Published:** 2018-10-04

**Authors:** Yuto Hozaka, Hiroshi Kurahara, Yuko Mataki, Yota Kawasaki, Satoshi Iino, Masahiko Sakoda, Shinichiro Mori, Kosei Maemura, Hiroyuki Shinchi, Shoji Natsugoe

**Affiliations:** a Department of Digestive Surgery, Breast and Thyroid Surgery, Graduate School of Medical Sciences, Kagoshima University, Japan; b Health Sciences, Kagoshima University, Japan

**Keywords:** Drainage, Abscess, Step-up approach, Embolization, Case report

## Abstract

**Introduction:**

Colorectal complications including penetration and perforation in acute pancreatitis often become severe and fatal. Effective drainage is pivotal for successful treatment. We present a case of large retroperitoneal abscess with colonic necrotizing perforation due to severe acute pancreatitis treated with video-assisted retroperitoneal debridement (VARD) in a step-up approach.

**Presentation of case:**

A 31-year-old man was admitted to a general hospital with a diagnosis of severe acute pancreatitis. Ten days after onset, he was referred to our hospital for more intensive treatment. On day 16, he experienced melena and shock, and embolization of the three straight arteries of the descending colon was performed. On day 30, percutaneous drainage was performed for large retroperitoneal abscess. On day 36, ileostomy was performed because the drained pus from the retroperitoneal abscess became feces-like. On day 58, VARD was performed to treat the refractory retroperitoneal abscesses causing high systemic inflammation due to insufficient drainage. On day 85, fluoroscopic examination showed disappearance of the abscess cavity. He was transferred to the previous hospital on day 89.

**Discussion:**

Colonic perforation due to severe acute pancreatitis often causes sepsis and fatal condition of patients, and drainage of the retroperitoneal abscesses via laparotomy is thought to be highly invasive and risky. VARD enables radical necrosectomy and drainage less invasively.

**Conclusions:**

VARD enabled less invasive treatment for patients with large retroperitoneal abscess due to colonic necrotizing perforation in severe pancreatitis.

## Highlights

• Severe acute pancreatitis with necrotizing colonic perforation is refractory and the mortality is high.
• Step-up approach for severe acute pancreatitis with infectious walled-off necrosis has been increasingly used.
• We performed percutaneous drainage, ileostomy, and video-assisted retroperitoneal debridement (VARD) as a step-up approach.
• VARD enable sufficient washing of the abscess and radical debridement of the necrotic tissues under direct view.

## 1 Introduction

Treatment for severe acute pancreatitis with colonic perforation is difficult and the mortality is high [[1], [2], [3]]. In most of previous reports, colon resection and ileostomy/colostomy were performed simultaneously. However, these procedures are highly invasive and likely to cause refractory wound infection in patients with marked intra-abdominal adhesions and large abscesses. We present a case of severe acute pancreatitis with large retroperitoneal abscess due to colonic necrotizing perforation treated by video-assisted retroperitoneal debridement (VARD) in a step-up approach. This case report has been reported in line with the SCARE criteria [4].

## 2 Presentation of case

**Fig. 1:**
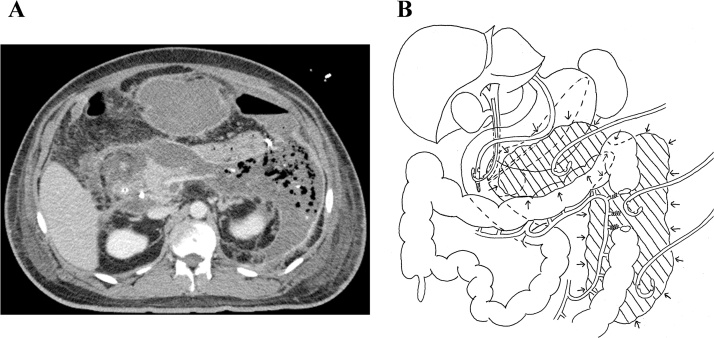
**Retroperitoneal abscess and drainage.** A: CT scans showed wide range of retroperitoneal abscess formation. B: Ultrasound-guided percutaneous abdominal drainage was performed for 3 areas of retroperitoneal abscesses.

**Fig. 2:**
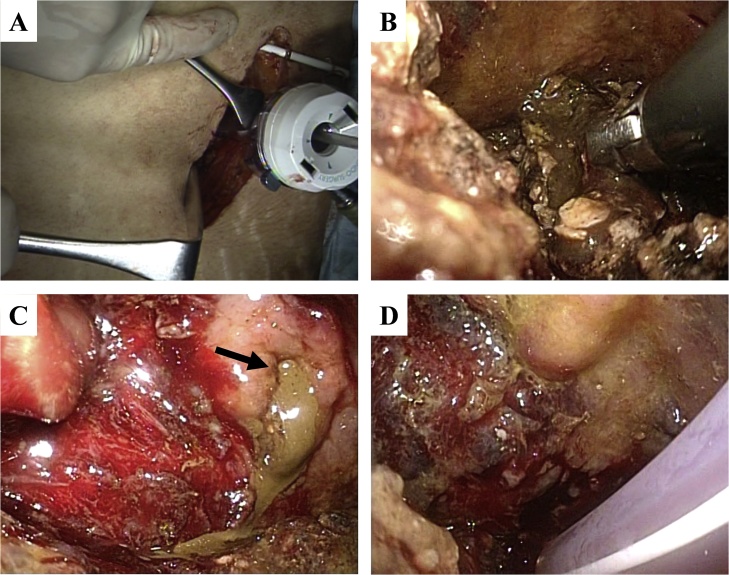
**Findings during surgery.** A: Retroperitoneal abscess was approached using percutaneous drainage catheter. B: Infected necrotic tissues were removed under direct vision. C: After the necrotic substances were removed, leakage of feces from the retroperitoneal perforated site of the colon was observed (arrow).

A 31-year-old man was admitted to a general hospital with the diagnosis of severe acute pancreatitis due to fallen gallstones. Pancreatic and biliary stents were placed by emergency endoscopic retrograde cholangiopancreatography. Ten days after onset, he was referred to our hospital for more intensive treatment because his general condition deteriorated. Blood pressure on admission was 155/100 mmHg, heart rate was 120 bpm, body temperature was 38.2 °C, and SpO_2_ was 100% (nasal high flow, 40 L/min; FiO_2_, 0.5). Body mass index (BMI) was 34.3. The laboratory data obtained at admission showed leukocytosis (white blood cells, 18,650/mm^3^), C-reactive protein elevation (32.6 mg/dL), D-dimer elevation (7.7 μg/dL), and low serum albumin levels (1.8 g/dL). Computed tomography (CT) images obtained on admission showed inflammation spreading to the lower margin of the left kidney. We administered intensive care management including antibiotics and proteolytic enzyme inhibitors. On day 16, emergency CT and angiography were performed because he had melena and developed shock. Transcatheter arterial embolization (TAE) of the three straight arteries of the descending colon was performed. On day 30, CT scans showed wide range of retroperitoneal abscess formation (Fig. 1A). Ultrasound-guided percutaneous abdominal drainage using three pig tail catheters (6-Fr) was performed. The first catheter was inserted in the abscess on the front of the pancreas, the second was inserted in the abscess around the splenic flexure of the left colon, and the third was inserted in the abscess around the descending colon (Fig. 1B). On day 36, ileostomy was performed because the appearance of drained pus from the abscesses became feces-like. Laparoscopic ileostomy was performed at the lower right abdomen. Laparoscopic intra-abdominal exploration was difficult because of marked obesity and firm adhesion between the abdominal wall and greater omentum. After ileostomy, his general condition improved temporarily. However, a highly inflammatory state with septic conditions and high fever occurred again. On day 58, VARD was performed to treat the refractory inflammatory condition due to insufficient drainage. First, the patient was placed in the right lateral decubitus position. A 6-cm skin incision was made on the foot side of the catheter (the second one) inserted from the middle axillary line of the ninth intercostal space to the abscess around splenic flexure of the left colon. The abscess cavity was approached from the tenth intercostal space, and the content of the abscess was removed as much as possible under direct vision. Then, a 12-mm port was inserted in that hole (Fig. 2A). The abscess cavity was fully observed using the scope inserted through the port, and infected necrotic tissues were removed through the open window of the tenth intercostal space (Fig. 2B). After the necrotic substances were removed, leakage of feces from the retroperitoneal perforated site of the colon was observed (Fig. 2C). Next, the patient was placed in the supine position. A 3-cm skin incision was made on the foot site of the catheter (the first one) inserted from the upper abdominal midline into the abscess on the front of the pancreas. Then, the catheter was replaced by a 12-mm port. The abscess cavity was observed using the scope. Then, the contents, which were pus and infectious hematoma, were removed under the scopic view. After washing with 10 L of saline solution, three silicone drains (10-mm) were placed in the washed cavities; two drains were placed in the left side cavity (dorsal side of the perforated colon and the descending colon) and one drain was placed in the middle cavity on the front of the pancreas (Fig. 2D). The operative time was 197 min, and the bleeding volume was 410 mL.

**Fig. 3:**
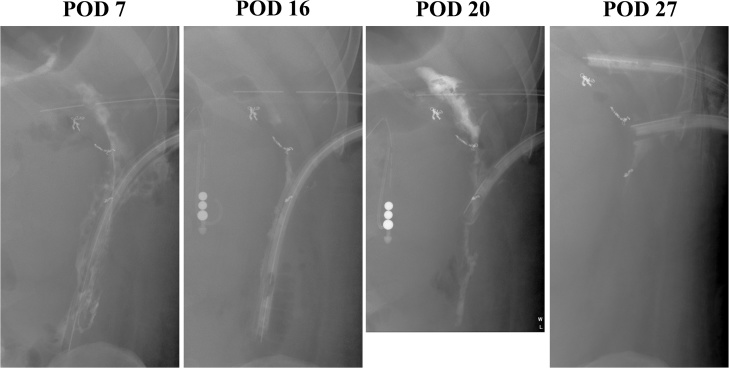
**Course of treatment for the abscesses.** The abscess cavities gradually contracted and disappeared.

On postoperative day 7, his inflammatory state and general condition markedly improved. On postoperative days 7, 16, and 20, fluoroscopic imaging examinations of the drains were performed to confirm contraction of the abscess cavity. On postoperative day 27, fluoroscopic imaging showed disappearance of the abscess cavity (Fig. 3). Drains in the upper abdominal midline and dorsal side of the descending colon were removed on the same day. The patient was transferred to the previous hospital on day 89 after onset (postoperative day 31). Thereafter, size of the remaining drain placed dorsal side of the perforated colon was reduced gradually, and drainage of pus nearly disappeared. Two months later, he was discharged from the hospital with 7 Fr Nelaton catheter and got back to his work. Nowadays, he is continuing outpatient visit once a month for exchange of the catheter.

## 3 Discussion

An infectious walled-off necrosis (WON) in severe acute pancreatitis often require interventional treatment [[5], [6], [7]]. A step-up approach, as compared with open necrosectomy, has been reported to reduce the rates of major complications and death [8]. The first step of this approach is percutaneous or endoscopic drainage. If there was no clinical improvement, VARD or endoscopic transluminal necrosectomy is performed as the second step [9]. VARD enables lumen observation of the abscess cavities and radical necrosectomy without invasive laparotomy. A skin incision is made safely under the guidance of the drainage catheter inserted through retroperitoneal rout. VARD requires less destruction of the abdominal wall, has shorter operative times, alleviate postoperative pain, and reduce major postoperative complications compared with open necrosectomy [[9], [10], [11]]. However, interventional treatment should be performed carefully because there is no anatomical indicator in the abscess cavity. Because the boundary between the necrotic and the normal tissues is unclear during the early onset stage, bleeding and normal tissue damage could occur easily. Therefore, it should be performed 4 weeks or later after onset so that necrotic foci are sufficiently encapsulated [8,12,13].

In the present case, a large amount of bleeding occurred during conservative treatment for severe acute pancreatitis, resulting in hemorrhagic shock. Incidence of colorectal complications in severe acute pancreatitis is 15–27% [3,14], and the mortality of patients with colorectal complications is as high as 17–67% [15]. Bleeding was treated with emergency TAE. However, necrotic perforation of the colon and a wide range of retroperitoneal abscesses occurred 12 days later. Percutaneous drainage based on the step-up approach was performed. Even after ileostomy and percutaneous drainage of the abscess cavities, the highly inflammatory condition did not improve due to insufficient drainage. Drainage of the retroperitoneal abscesses via laparotomy or simultaneous resection of the perforated colon was thought to be highly invasive and risky because of the marked intra-abdominal adhesions, severe systematic inflammation, and obesity (BMI, 34.3). Therefore, we selected VARD as a less invasive treatment.

Although PubMed was searched for articles published in English from January 1950 to September 2017 that included the terms “pancreatitis,” “TAE,” and “colonic perforation,” there were no report of colonic perforation after TAE in severe pancreatitis. Lim et al. reported a case of retroperitoneal bleeding and colonic fistula after VARD for infected necrotizing pancreatitis [16]. They performed TAE of the splenic artery and ileostomy. After the ileostomy was closed 12 months later, the patient developed refractory colo-cutaneous fistula. They treated by endoscopic clips and histoacryl glue injection instead of colectomy. It has been reported that colonic perforation due to ischemia after embolization could occur [17]. In the present case, ischemia due to embolization in addition to inflammation due to severe pancreatitis may have caused perforation of the colon near the splenic flexure. Because the transverse colon and splenic region of the left colon are close to the pancreas, those are likely affected by inflammation due to severe pancreatitis [18,19]. It has been speculated that inflammatory substances, which are rich in pancreatic enzymes, reach the mesocolon and cause thrombosis and pericolitis, and that intestinal tract ischemia is the cause of colonic perforation in severe pancreatitis [19]. Aldridge reported that resection of the perforated colon is required in severe acute pancreatitis because it cannot be expected to have natural closure, however, the mortality is very high [20]. In most previous reports, pancreatitis-related colonic perforation was treated with simultaneous colon resection and ileostomy/colostomy. VARD enabled removal of the wide range of retroperitoneal abscesses and placement of silicon drains at appropriate position under direct view, that led to rapid recovery from the severe inflammatory state without simultaneous colectomy. We need to reevaluate the perforated colon by colonoscopy and gastrograffin enema examinations to decide treatment strategy several months later. Nevertheless, even resection of the perforated colon may be performed more safely in good general condition.

## 4 Conclusions

In addition to temporary ileostomy, VARD could be an effective treatment method for the wide range of retroperitoneal abscesses due to colonic perforation in patients with severe acute pancreatitis.

## Conflicts of interest

The authors declare that they received no funding for this work, and they have no conflict of interest.

## Funding source

The authors did not have any sponsors of this work.

## Ethical approval

This study was approved by the ethics review board of Kagoshima University (No. 25-39).

## Consent

Written informed consent was obtained from the patient for publication of this case report and accompanying images.

## Author contribution

Yuto Hozaka, Hiroshi Kurahara: study concept and drafting the manuscript.

Yuko Mataki, Yota Kawasaki, Satoshi Iino, Masahiko Sakoda: acquisition and analysis of data.

Shinichiro Mori, Kosei Maemura, Hiroyuki Shinchi, Shoji Natsugoe: revising the manuscript.

All authors read and approved the final manuscript.

## Registration of research studies

Name of the registry: Successful treatment for severe pancreatitis with colonic perforation using video-assisted retroperitoneal debridement: a case report.

UIN: researchregistry4339.

## Guarantor

Hiroshi Kurahara is guarantor who accept full responsibility for the work and/or the conduct of the study, had access to the data, and controlled the decision to publish.
